# Poxviral Strategies to Overcome Host Cell Apoptosis

**DOI:** 10.3390/pathogens10010006

**Published:** 2020-12-23

**Authors:** Chathura D. Suraweera, Mark G. Hinds, Marc Kvansakul

**Affiliations:** 1 Department of Biochemistry and Genetics, La Trobe Institute for Molecular Science, La Trobe University, Melbourne, VIC 3086, Australia; 18196819@students.latrobe.edu.au; 2 Bio21 Molecular Science and Biotechnology Institute, The University of Melbourne, Parkville, VIC 3052, Australia

**Keywords:** Bcl-2, pox virus, apoptosis

## Abstract

Apoptosis is a form of cellular suicide initiated either via extracellular (extrinsic apoptosis) or intracellular (intrinsic apoptosis) cues. This form of programmed cell death plays a crucial role in development and tissue homeostasis in multicellular organisms and its dysregulation is an underlying cause for many diseases. Intrinsic apoptosis is regulated by members of the evolutionarily conserved B-cell lymphoma-2 (Bcl-2) family, a family that consists of pro- and anti-apoptotic members. Bcl-2 genes have also been assimilated by numerous viruses including pox viruses, in particular the sub-family of chordopoxviridae, a group of viruses known to infect almost all vertebrates. The viral Bcl-2 proteins are virulence factors and aid the evasion of host immune defenses by mimicking the activity of their cellular counterparts. Viral Bcl-2 genes have proved essential for the survival of virus infected cells and structural studies have shown that though they often share very little sequence identity with their cellular counterparts, they have near-identical 3D structures. However, their mechanisms of action are varied. In this review, we examine the structural biology, molecular interactions, and detailed mechanism of action of poxvirus encoded apoptosis inhibitors and how they impact on host–virus interactions to ultimately enable successful infection and propagation of viral infections.

Apoptosis is a form of programmed cell death activated in response to either extracellular (extrinsic apoptosis) or intracellular (intrinsic apoptosis) stimuli [1]. Playing a pivotal role in development and tissue homeostasis in multicellular organisms apoptosis selectively removes unwanted, damaged, or pathogen infected cells [2]. Apoptosis probably arose initially as a defense mechanism against pathogens and was subsequently adapted for additional purposes such as control of tissue morphogenesis during development [3,4]. While necessary for homeostasis and other regulatory roles, subversion of apoptosis underlies an array of diseases including cancers and autoimmune diseases [5]. The importance of regulating host cell death and immune responses has triggered the assimilation of many apoptotic regulatory genes by viruses. Poxviruses, in particular, have captured numerous genes for manipulating apoptosis such as viral Bcl-2 (vBcl-2) homologs, serpin protease inhibitors, dsRNA inhibitors, NF-κB inhibitors [6,7], or Interferon (IFN) inhibitors [8,9,10].

Metazoans share homologous genes that regulate both intrinsic and extrinsic apoptosis such as Bcl-2 proteins, caspases, and adaptor proteins [4]. Both intrinsic and extrinsic apoptosis initiation are governed by the activation of cysteine aspartyl proteases (caspases) that degrade intracellular targets [11]. However, there are significant differences in how the caspase cascade is initiated in intrinsic apoptosis compared with extrinsic apoptosis.

Extrinsic or death receptor mediated apoptosis initiates when death ligand (Fas L/TNF-α)/TNF related apoptosis inducing ligands (TRAIL) bind and oligomerize tumor necrosis factor (TNF) superfamily receptors at the cell surface. The TNF superfamily receptors bear cytoplasmic death domains (DD) as protein interaction modules [12] that recruit intracellular ligands through protein–protein interactions between receptor DDs and ligand DDs. TNF-receptor superfamily signaling is complex and the resultant biological outcome is dependent on the extracellular ligand bound and activates both apoptotic and non-apoptotic pathways [13]. Adaptor proteins such as FADD (Fas associated death domain protein) or TRADD (TNF receptor associated death domain protein) are recruited via their DDs once TNF receptors are activated to form the multiprotein death inducing signaling complex (DISC). DISC formation is essential for downstream activation of the caspase cascade [12,14] and there are numerous post-translational controls on their activation [15]. FADD and TRADD in addition to their DDs bear structurally similar death effector domains (DED) that interact homotypically with the DED of pro-caspase-8 to recruit it to the DISC complex. At the DISC, inactive pro-caspase-8 undergoes proteolytic cleavage to release p18/p12 domain to form the active caspase-8 homodimer that subsequently proteolytically activates the executioner caspases (caspase-3, -6, and -7). In turn, caspase-8 activation at the death receptor is modulated by heterodimerization with the cellular FLICE (FADD-like IL-1_converting enzyme)-like inhibitory protein (cFLIP) though its N-terminal DED domains. Interaction with cFLIP, a protein that is structurally related to pro-caspase-8 but bearing a catalytically inactive caspase-like domain in addition to its two N-terminal DED modules prevents caspase-8 homodimerization, a key step in forming active caspase [15]. Inhibition of pro-caspase-8 activation leads to cell survival and viruses have assimilated genes that mimic cFLIP to inhibit this [16]. In addition to activating the caspases, active caspase-8 cleaves the cellular BH3 interacting domain (Bid) protein to activate this BH3-only protein for Bcl-2 initiated apoptosis and thus links extrinsic apoptosis with intrinsic apoptosis [17]. Furthermore, cellular inhibitors of apoptosis proteins (cIAPs) are able to control the extrinsic apoptosis pathway by regulating DISC formation and under certain conditions trigger TNF-α to initiate the activation of nuclear factor κB (NF-κB) [18].

Intrinsic apoptosis is initiated by intracellular signals and is primarily regulated by Bcl-2 family genes [1,5]. Though not all metazoans share the Bcl-2 genes [19], they generally have been well conserved from the earliest metazoans such as sponges, placozoans, and cnidarians [19,20,21,22] to worms [23], fish [24], and humans [25]. The Bcl-2 family is split into pro- and anti-apoptotic Bcl-2 members that all share up to four conserved sequence regions or Bcl-2 homology motifs (BH1-BH4), and are central to their action [19,26]. The pro-apoptotic Bcl-2 proteins are further subdivided into two groups, the multi-motif pro-apoptotic Bcl-2 (whose members include Bax, Bak, and Bok) that are closely related in sequence and structure to the pro-survival Bcl-2 proteins, and the BH3-only proteins (Bad, Bid, Bim, Bik, Bmf, Hrk, Noxa, and Puma) that are phylogenomically more distant [27]. Several models have been proposed for the action of the BH3-only proteins, but their main role appears to be to inhibit the pro-survival Bcl-2 proteins, though they may also activate some pro-apoptotic members [28]. Structural studies have shown that the BH3 region of pro-apoptotic proteins binds in a groove provided by the pro-survival protein [27]. Notwithstanding the unresolved issue regarding interaction with Bax or Bak, BH3-only proteins are major apoptosis inducers that are activated in response to various cellular insults and initiate the cell death process, leading to Bak and Bax oligomerization at the mitochondria outer membrane (MOM) and its permeabilization (MOMP) [29]. MOMP leads to the release of cytochrome *c* and other apoptosis inducing factors from the mitochondria inter membrane space that activates the caspase cascade that ultimately leads to cell death [30]. Other roles have also been ascribed to the Bcl-2 proteins including autophagy [31,32] and cytosolic Ca^2+^ regulation [33].

Since apoptosis plays an important front-line defense mechanism against invading pathogens [1,26], viruses have evolved multiple strategies to block host cell apoptosis [7,34] to enable their successful infection and replication [35]. Most large DNA viruses including the poxviruses utilize protective responses during infection to keep the host-cells alive by molecular mimicry, allowing them to produce structural, functional, and sequence homologs of cellular pro-survival proteins including Bcl-2 proteins to overturn host cell apoptosis [7,34]. Pro-survival genes including those of the Bcl-2 family have been acquired by viruses [2,19]. While Bcl-2 genes are not the only pro-survival factors in viral genomes, they are probably the most well understood at a molecular level [36]. The pro-survival Bcl‑2 proteins exist as a globular helical bundle comprising seven or eight alpha helices, with helices α2-α5 forming the canonical hydrophobic ligand binding groove that provides the interaction site for BH3 motifs of pro-apoptotic Bcl-2 proteins [2]. Not all Bcl-2 mimics share significant sequence similarity with mammalian Bcl-2 proteins, indeed, some have very low shared identity (<10%), which makes them difficult to identify from their sequence alone [37,38,39]. For example, the Bcl-2 homolog of Epstein Barr Virus (EBV) BHRF1 has 17.5% shared identity with human Bcl-x_L_ with homologous residues clustering in BH motifs, whereas the myxoma virus homolog M11L shares only 9.6% and features no obvious BH motifs [37], however, both vBcl-2 homologs adopt the canonical Bcl-2 fold, obstruct premature host cell death, and are critical for successful infection and proliferation [40]. 

Subversion of host cell apoptosis by viral Bcl-2 homologs employs the interactions between pro-survival vBcl-2 proteins and cellular pro-apoptotic Bcl-2 proteins Bax, Bak, or BH3-only proteins, in a similar mode of action of their cellular counterparts [41] (Figure 1). These interactions have been widely studied and affinity measurements reported and the major finding is that there are significant variations in the BH3 binding profile of the vBcl-2 proteins [41]. The structures of a number of vBcl-2 proteins either ligand free or as complexes with their potential cellular targets were determined (Figure 1). These findings suggested that some viruses need apoptosis to escape the host cell and thus apoptosis is only delayed, not prevented. Other viruses need to block the apoptosis by selectively mimicking and supplementing the action of particular pro-survival Bcl-2 proteins, which are important during viral infection and proliferation.

The earliest vBcl-2 homologs identified were those discovered by the presence of their characteristic BH sequence motifs and these regions were confirmed to be vital for their function [42]. Viral Bcl-2 members included in this group are those of E1B19K from adenovirus [43,44], the herpesviruses (*herpesviridae*) Epstein Barr virus (EBV), BHRF1 [45,46], Kaposi sarcoma virus, KsBcl2 [47], turkey herpes virus vNR13 (a viral Bcl-B ortholog) [48], Herpesvirus saimiri ORF16 [49], and murine γ-herpes virus 68/M11 [50]. Bcl-2 genes have also been identified in *asfarviridae* (African swine fever virus, ASFV A179L) [51,52], and *iridiviridae* (Grouper Iridovirus GIV66 [53]). Saliently, *poxviridae* members such as vaccinia virus (VACV) have been shown to bear Bcl-2 genes (VACV F1L) [38,54], however, the lack of identifiable primary sequence identity with cellular Bcl-2 proteins for many of the poxvirus encoded genes hampered their identification as *bona fide* Bcl-2 proteins.

Poxviruses have relatively large and complex genomes when compared to other viruses, and employ multiple strategies for modulating host-cell apoptosis [7]. Frequently, poxviruses contain multiple Bcl-2 mimics (e.g., VACV N1L and F1L are both Bcl-2 mimics) that interfere with Bax-Bak regulated apoptosis that attests to the importance of manipulating this pathway. Other strategies employed by poxviruses include TNF receptor homologs such as CrmB, CrmC, CrmD, and CrmE [55], Serine protease inhibitors (CPXV CrmA, VACV B13) [56,57], Golgi anti-apoptotic protein GAAP [58], double stranded RNA (dsRNA) induced apoptosis (e.g., VACV E3, MYXV M029, SPV032) [59,60] and Cu–Zn–Superoxide dismutase (SOD) induced apoptosis inhibition (M131, S131) [61]. These strategies are summarized in Figure 2. However, there is significantly less structural and interaction data available for these non-Bcl-2 mimics. Apart from potential health risk from poxviruses, current research is focusing on various immunomodulatory strategies encoded by various poxviruses against host immune systems [7,62]. In this review, we discuss details of the poxvirus Bcl-2 modulated host apoptosis inhibitory strategies to overcome the cellular apoptosis response.

## 1. Pox Virus Inhibition of Host Intrinsic Activated Apoptosis with Bcl-2 Homologs

*Poxviridae* are a sizeable and diverse group of viruses that infect both vertebrates and invertebrates and are subdivided into the *entomopoxviridae*, which infect invertebrates such as insects, and *chordopoxviridae*, which infect vertebrates [63]. Ten genera of *poxviridae* are currently identified and classified under *chordopoxviridae* [63]. These are orthopoxvirus, capripoxvirus, cervidpoxvirus, suipoxvirus, leporipoxvirus, mollusicpoxvirus, yatapoxvirus, avipoxvirus, crocodylidpoxvirus, and parapoxvirus. Among these phyla, orthopoxvirus, molluscipoxvirus, yatapoxvirus, and parapoxvirues have been shown to infect humans and cause disease [64]. For example, monkeypox virus is an orthopoxvirus and is classified as an emerging zoonotic disease that could have a potentially significant impact on human health [65].

Poxviruses are large linear double stranded DNA viruses that contain 135–360 kbp, which encode up to 328 open reading frames (ORFs) [66] and exclusively replicate within the cytoplasm of the infected cells [63]. Perhaps the two most well-known examples of the pox family are variola virus (VARV), the causative agent responsible for smallpox and vaccinia virus (VACV), the vehicle for delivery of the smallpox vaccine [67]. VARV and VACV are closely related orthopoxviruses [68] that bear multiple immunomodulatory genes including Bcl-2 homologs. [38,69,70,71,72]. Almost all poxviridae of the chordopox families encode Bcl-2 like proteins and significantly no entomopox viruses have yet been identified with Bcl-2 homologs in their genomes. This observation is likely due to differences in Bcl-2 mediated apoptosis in invertebrates compared to vertebrates [1,19]. Here, we review the state of knowledge on the Bcl-2 genes in the chordopox viruses and their structural biology, interactions with the cellular pro-apoptotic Bcl-2 members, and mechanisms of action to block host apoptosis, thus shedding light on how chordopox viruses successfully infect and replicate inside host cells.

**Figure 1:**
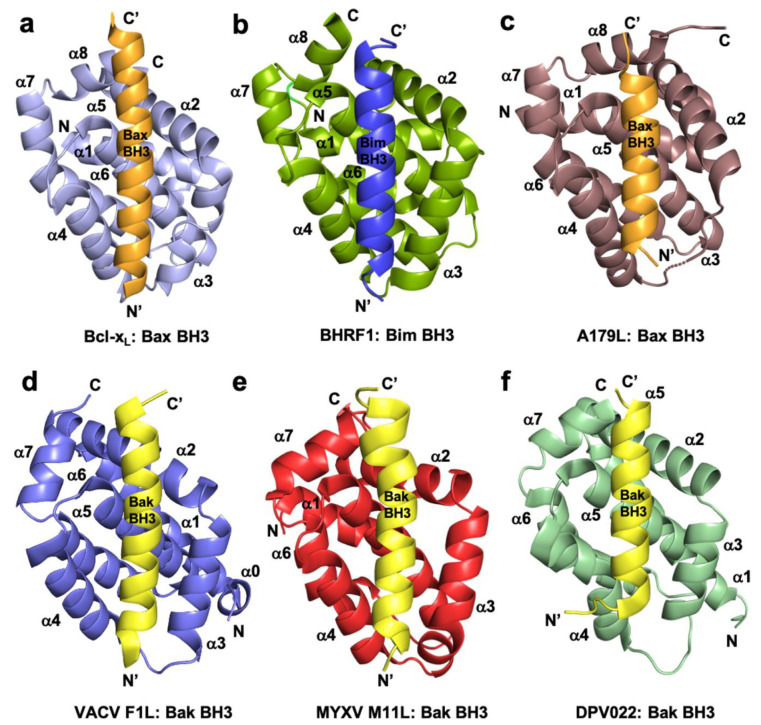
Cartoon representation of crystal structures of poxviral encoded Bcl-2 proteins and comparison with cellular Bcl-xL and other viral Bcl-2 structures. (**a**) Human Bcl-xL (light blue) in complex with Bax BH3 (tv orange) [73]. All helices are labelled as α1-α7/8 and the view is into the canonical ligand binding groove formed by helices α2-α5. (**b**) EBV BHRF1 (split pea) in complex with Bim BH3 (blue) [45], (**c**) ASFV A179L (dirty violet) in complex with Bax BH3 [52] (tv orange), (**d**) VACV F1L (slate) bound to Bak BH3 (yellow) [54], (**e**) MYXV M11L (red) bound to Bak BH3 (yellow) [37], and (**f**) DPV022 (wheat) in complex with Bak BH3 (yellow) [74]. The orientations depicted in (**b**)–(**f**) are identical to that in (**a**).

### 1.1. Orthopoxviruses

VACV, the prototypical member of *orthopoxviridiae,* bears multiple proteins that have either been established or predicted to have structures similar to the Bcl-2-fold [75]. Examples of structures of this class of protein have been given in Figure 3. 

**Figure 2:**
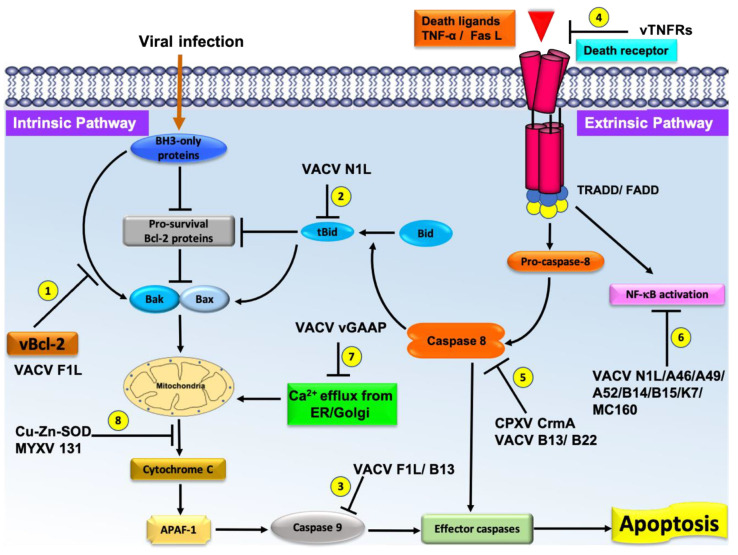
Schematic diagram of the major pathways of apoptosis and the virus encoded effector molecules that modulated them. All major apoptosis inhibition mechanisms utilized by poxviruses are numbered as 1–8. The cellular intrinsic apoptosis pathway is initiated in the event of viral infection, leading to activation of BH3-only proteins in the cytoplasm. These BH3 proteins interact with cellular pro-survival Bcl-2 proteins to neutralize their activity or directly interact with cellular pro-apoptotic Bcl-2 proteins, releasing them to oligomerize at the mitochondrial outer membrane, leading to membrane permeabilization. Release of cytochrome c and other factors from mitochondria activates the downstream events of intrinsic apoptosis such as APAF-1 activation. Virally encoded Bcl-2 proteins mimic the action of cellular pro-survival Bcl-2 proteins to block apoptosis by a variety of mechanisms. (1) VACV F1L interacts with BH3-only proteins to block their activity and subsequent activation of mitochondrial mediated apoptosis. (2) VACV N1L interacts with cellular BH3-only protein Bid to inhibit the downstream activation of apoptosis. (3) VACV F1L/VACV B13 interacts with caspase-9 and suppress the activation of the caspase cascade and subsequent cell death. (4) Poxvirus encoded vTNFRs mimic cellular TNFR1/2 and block the initiation of extrinsic apoptosis. (5) Poxvirus encoded CPXV CrmA/ VACV B13/B22 antagonize active caspase-8 and downstream activation of intrinsic or extrinsic apoptosis. (6) VACV N1L/A46/A49/A52/B14/B15/K7/MC160 inhibit the activation of NF-κB activation, (7) VACV GAAP blocks the Ca^2+^ efflux from Golgi/ER, and (8) Cu–Zn–SOD encoded by MYXV inhibits the MOMP and subsequent activation of intrinsic apoptosis.

**Figure 3:**
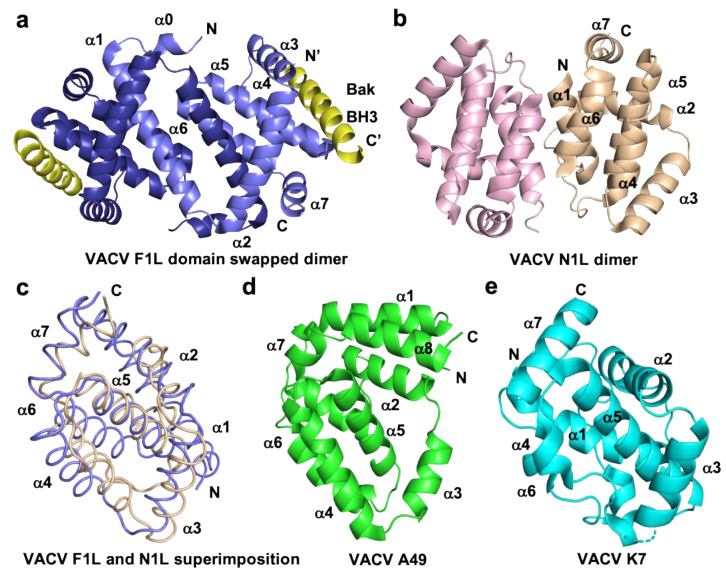
Cartoon representation of different topologies of VACV Bcl-2 homologs. (**a**) Crystal structure of the dimer formed by VACV F1L (slate) in complex with Bak BH3 (yellow) where α1 helix of one protomer is swapped with α1 helix of a neighboring protomer, (**b**) dimer interface formed by VACV N1L (beige), where α1 helix of one protomer interact with α6 of the adjacent protomer in crystal contact. (**c**) Structural superimposition of cartoon tube representation of VACV F1L and VACV N1L as in a view of hydrophobic binding groove made by α2-α5 facing to front. (**d**) Cartoon representation of A49 (green) and (**e**) structure of K7 (cyan). Helices are labelled α1–α7 in (**a**)–(**e**).

VACV F1L was the first identified Bcl-2 like protein linked to mitochondria associated apoptosis inhibition in vaccinia virus [76], but shares no recognizable sequence identity with any mammalian Bcl-2 family members. Functional studies of VACV F1L revealed that it is an essential element for survival of virus infected cells and prevented staurosporin induced cell death and subsequent cytochrome *c* release from mitochondria in Jurkat cells [76]. In comparison, an *f1l* deletion mutant, VV811, underwent apoptosis and expression of F1L prevented all post-mitochondrial events [76,77]. Similar to other pro-survival Bcl-2 proteins, VACV F1L was also shown to localize to mitochondrial membranes through its hydrophobic C-terminal residues [76]. Biochemical interaction studies of VACV F1L revealed that it has a highly selective BH3 binding profile and is only able to bind to peptides that span the BH3 regions from Bim, Bak, and Bax with sub micromolar affinities [38]. Interaction of VACV F1L with Bim is key to its pro-survival activity [54]. Mutagenesis of the F1L binding groove residue, A115W, hindered the interactions with Bim_L_, but not with Bak and the mutant was unable to prevent host cell apoptosis [54]. In addition, F1L prevented Bak and Bax homo-oligomerization and subsequent cell death [77], in part by replacing the activity of Mcl-1 [78]. The crystal structure of F1L featured the conserved Bcl-2 fold with seven α-helices where helices α2–α5 form the canonical ligand binding groove, but the overall fold was a dimer featuring an unusual domain swapped configuration where the α1 helix of two neighboring protomers were swapped (Figure 3). In contrast to F1L, mammalian pro-survival Bcl-2 protein possesses eight α-helices that feature an additional short helix near the C-terminus. In addition to the domain swapped dimer conformation, F1L features a unique N-terminal extension spanning residues 1–56. It was previously reported that this extension harbors caspase-9 inhibitory activity [79] and was predicted to be helical [80]. However, a subsequent study demonstrated that the N-terminal extension was intrinsically disordered and did not contribute to apoptosis regulation [38,81]. More recent functional analysis of this N-terminal region suggested an inflammasome inhibitory function via direct interaction with NLRP1 [82], which is important for initiating innate immune responses against invading pathogens [83] (Figure 2). VARV also encodes an F1L homolog, VARV F1L, and although it has an almost identical structure and sequence to VACV F1L [39], it differs functionally from VACV F1L. VARV F1L inhibits host apoptosis through interacting with only Bid, Bak, and Bax but does not bind Bim (Table 1). Compared to VACV F1L, VARV F1L only inhibits Bax mediated apoptosis but does not inhibit apoptosis via Bak [39].

Other orthopox viruses have not been as well studied and the data available on their vBcl-2 function are much more limited in scope. Ectromelia virus is the causative agent of mousepox and expresses an F1L homolog, EMV025. EMV025 was found to interact with Bak, Bax, and Bim, and blocked the host intrinsic apoptosis pathway by sequestering Bak [84,85].

VACV also bears a second apoptosis inhibitory Bcl-2 like protein in its genome in addition to F1L, the 117-residue protein N1L. N1L has been shown to interact with several cellular pro-apoptotic proteins including Bak, Bid, and Bim with high affinity (Table 1), similar to that observed previously for Bcl-x_L_ [72,86]. In contrast to other vBcl-2 proteins, N1L is localized in the cytosol but not mitochondria and lacks the C-terminal hydrophobic region which in Bcl-2 proteins targets the outer mitochondrial membrane [72]. The crystal structure of VACV N1L revealed that it adopted an overall Bcl-2 fold as a homodimer, where α1 and α6 formed the dimerization interface (Figure 3b) [72]. A structural comparison revealed that VACV F1L and N1L shared a similar structure (Figure 3c). However, functionally VACV N1L inhibits NF-κB signaling during infection as well as block the host intrinsic apoptosis pathway under specific conditions and these functions in N1L are mediated by two different independent sites [75,87]. This has been confirmed in transiently transfected N1L immuno-co-precipitated with cellular Bax, Bid, and Bad, where Bax was expressed by cellular transfection [86].

Regardless of their structural similarity to Bcl-2 proteins, Bcl-2 homologs do not necessarily involve manipulation of host cell apoptosis; most orthopox Bcl-2 homologs are associated with the regulation of host innate immune responses [8] through antagonizing the Toll Like Receptor (TLR) signaling network [88,89] (Figure 2).

VACV A46, which was initially predicted to be a member of the Bcl-2 family [89], and was subsequently shown to adopt a Bcl-2 fold comprising seven a-helices [90,91]. Notably, A46 does not harbor Bcl-2 like anti-apoptotic activity, instead, A46 is an inhibitor of the Toll/interleukin-1 receptor (TIR)-domain adaptor protein, which is crucial for triggering innate immune responses against invading pathogens [90,92,93]. Thus, VACV A46 particularly targets the TIR in a region known as the BB-loop, which is a well conserved short peptide sequence (30 residues) of TIR-domain proteins [94] that has no shared sequence identity with BH3 motifs and blocks the interactions between receptor and adaptor [91] and downstream activation of NF-κB signaling [90]. The crystal structure of A46 showed that it exists as a homodimer with a Bcl-2 fold similar to that seen in VACV N1L [72], A52 [95], B14 [95], and K7 (Figure 3) [91,96], where the α2 helix of one protomer interacts with the α6 helix of the neighboring protomer to form the dimer interface [91,95,97]. A46 was shown to interact with the TIR motif of adaptor protein MAL (MyD88-adaptor-like protein) with 13 uM affinity via the α1 helix of A46, and mutational analysis showed that the mutant E97A reduced the affinity by around 36-fold and K88A reduced by 4-fold, in which E97 played a significant role [91]. Recent data revealed that A46 interferes with the formation of filaments from TIR domains of both MAL and MyD88, two TLR adaptor proteins whose filaments trigger the early activation of NF-κB. Mutagenesis data mapped the interaction site of A46 with MAL/MyD88 filaments to a region spanning a-helices 1 and 7 as well as the flexible C-terminus, thus providing a mechanistic insight into A46 mediated inhibition of NF-κB activation [98].

VACV A52 and B14 are intracellular 23 kDa and 17 kDa proteins, respectively, which were predicted to have Bcl-2 folds by secondary structure prediction [89]. VACV A52 and B14 express early in the infection cycle and are important virulence factors that function by inhibiting the activation of NF-κB signaling [95,99] (Figure 2). Structural and solution analysis of VACV A52 and B14 (Figure 3d,e) showed them to homodimerize both in-vitro as well as in the crystal structure as previously reported for VACV N1 and A46 [95]. Neither A52 nor B14 form a hydrophobic binding groove that is required for interaction with the BH3 motif of pro-apoptotic Bcl-2 proteins [95]. An in-vivo transfection analysis reported that both A52 and B14 have a function in NF-κB signaling [95] though there is yet to be any structural analysis. Nevertheless, structure based phylogenetic analysis proposed that A52 and B14 are more similar to VACV N1 rather than Bcl-2 proteins, although they adopt a similar Bcl-2 fold [95]. 

VACV K7 is a 17 kDa intracellular Bcl-2 like protein that does not feature the canonical ligand binding groove unique to Bcl-2 proteins (Figure 3) and has been shown to interact with IRAK2 and TRAF6 in in vivo transfected assays to downregulate the activation of TLR-dependent NF-κB [100]. VACV K7 has been shown to interact with DEAD-box RNA helicase (DDX3), which plays an important role in the innate immune response [101] and binds VACV K7 with an affinity of 510 nM as determined from ITC experiments [101]. The crystal structure of VACV K7 in complex with DDX3 revealed that the DDX3 binding pocket on K7 is located in the region covered by the N-terminus of α1 helix and the α6 helix, and is largely unstructured [101]. These data suggest vaccinia virus has captured a Bcl-2 like gene and over time and adapted it for various immunomodulatory functions.

VACV A49 is a 19 kDa Bcl-2 like protein predominantly expressed in the cytosol and does not possess pro-survival function. A49 also does not feature a hydrophobic ligand binding groove, and structurally resembles MYXV M11L [102] (Figure 3c). However, homologs of A49 are only found in orthopoxviral genomes [103] and have been shown to inhibit NF-κB activation and translocation into the nucleus, with the A49 knockout virus unable to block the NF-κB activation [104]. Similar to VACV K7, A49 exists as a monomer in solution or in cells [104].

VACV encodes another inhibitor of intrinsic apoptosis, M1, which has not been structurally characterized [105], but is predicted to be an ankyrin-repeat like (ANK) protein. Mechanistically, M1L was shown to inhibit staurosporin-induced apoptosis, and co-immunoprecipitated with APAF-1: Caspase-9 complexes [105].

### 1.2. Leporipoxviruses

Leporipoxviruses (*Leporipoxviridae*) cause disease in rabbits and squirrels, and comprise four members with myxoma virus (MV) being considered the prototypical member of the genus [64]. MV is the causative agent of myxomatosis in European rabbits [106] and encodes a vBcl-2 like protein, M11L, which is localized to the MOM via its hydrophobic C-terminal region. The crystal structure of M11L showed that it adopts a monomeric Bcl-2 fold despite the lack of detectable primary sequence identity with cellular Bcl-2 proteins [37,107]. The structure of M11L features seven α-helices and a hydrophobic binding groove that engages with pro-apoptotic proteins, similar to that in other pro-survival proteins [37,107]. M11L displays high affinity toward the BH3-only protein Bim and was able to bind other pro-apoptotic proteins Bak [108], Bax, Bid, and Bmf with high to moderate affinity (Table 1) [37]. Cellular studies confirmed that, unlike VACV F1L, M11L subverts host cell apoptosis by primarily sequestering cellular Bak and Bax [37], but not Bim [54]. Myxoma virus infection initiates a rapid response from cellular Bax, which translocates to mitochondria [106]. Interestingly, expression of M11L could inhibit the Fas-ligand induced apoptosis in HEK293 cells and downregulate the subsequent caspase cascade in virus infected cells [108]. These data suggest that MYXV M11L may inhibit host apoptosis through both the intrinsic and extrinsic pathways.

### 1.3. Yatapoxviruses

*Yatapoxviridae* are primate specific poxviruses and two identified members, the tanapoxvirus (TANV) and yaba monkey tumor virus (YLDV), cause mild monkeypox like infections in humans [64]. Genome analysis of both TANV and YLDV revealed a putative Bcl-2 like protein 16L (TANV16L) [109] that shared a 98% sequence identity with each other. TANV16L displayed a broad range of interactions toward pro-apoptotic Bcl-2 proteins by binding to almost all BH3 interactors except Bok and Noxa (Table 1), and inhibited cell death induced by cellular Bax and Bax in a yeast model system [110]. Surprisingly, the crystal structures of TANV16L revealed both monomeric and domain swapped dimeric Bcl-2 configurations where two complexes of TANV16L, Bax BH3, and Bim BH3 crystallized as a domain swapped dimeric configuration and TANV16L: Puma BH3 complex crystallized in a monomeric Bcl-2 fold [110]. This was further validated by an analytical ultracentrifugation experiment showing TANV16L exists as mainly monomeric and dimeric forms with a minor component of homotetrameric species [110]. The dimeric topology of TANV16L is similar to that observed previously in vaccinia and variola virus Bcl-2 homologs [38,39], with the monomeric configuration only differing due to the α1 helix being folded back into the same protomer.

### 1.4. Parapoxviruses

*Parapoxviridae* are also known as epitheliotropic viruses and cause skin infections in humans. ORF virus is the prototypical member of this genus and commonly infects sheep, goats, and humans [111]. ORFV125 is a predicted anti-apoptotic Bcl-2 like gene encoded by the ORF virus [112]. Similar to other vBcl-2 proteins, ORFV125 lacks obvious Bcl-2 homologs, but antagonizes mitochondria mediated apoptosis and caspase activation in virus infected cells [113]. Immunoprecipitation data of ORFV125 revealed that it only engaged with a selective subset of pro-apoptotic proteins: Bim, Puma, Hrk, Bik, Noxa, and active Bax, but not Bak [114]. In contrast, recently reported affinity measurements of ORFV125 showed that it only binds to cellular Bax, Bak, Puma, and Hrk BH3 motif peptides with moderate sub micromolar affinities (Table 1) [115]. However, no interactions with the universal BH3 interactor Bim were detected. Crystal structures of ORFV125 revealed that it exists as a domain swapped dimer [115], similar to the previously reported VACV F1L [38], VARV F1L [39], DPV022 [74], and TANV 16L [110].

### 1.5. Capripoxviruses

Capripoxviruses are economically important pathogenic viruses that infect domestic ruminants such as sheep and goats. The difficult to control lumpy skin disease is a common disease of sheep caused by sheeppoxvirus, a prototypical member of capripoxvirus that causes significant economic loss by terminating wool production [116]. Sheeppox virus encoded SPPV14 is well characterized capripoxvirus anti-apoptotic vBcl-2 protein that adopts a monomeric Bcl-2 fold similar to M11L [117], and features a broad range of interactions binding all pro-apoptotic host Bcl-2 proteins [118] except Noxa (Table 1).

**Table 1:** Summary of poxvirus Bcl-2 homolog binding data. Binding affinities of poxviral Bcl-2 proteins measured by isothermal titration calorimetry (ITC) or surface plasmon resonance (SPR). All pox viral proteins were shown to interact with cellular Bak, Bax, and Bim except VARV F1L, which does not interact with Bim. This suggests that poxviruses primarily target cellular Bak and Bax or Bim inhibition and downregulate the subsequent activation of intrinsic apoptosis.

Binding Affinities (nM)									
Pro-apoptotic Protein	VACV F1L [38]	VARV F1L [39]	M11L [37]	TANV16L [110]	SPPV14 [117]	DPV022 [74]	FPV039 [119]	CNP058 [120]	ORFV125 [115]
Bak	4300	2640	50	38	48	6930	76	508	5802
Bax	1850	960	75	70	26	4040	76	326	682
Bok	N/A	N/A	N/A	NB	7580	N/A	NB	NB	NB
Bad	NB	NB	>1000	219	5197	NB	653	NB	NB
Bid	NB	3200	100	719	136	NB	2	50	NB
Bik	NB	NB	>1000	1250	1766	NB	30	NB	NB
Bim	250	NB	5	180	19	340	10	353	NB
Bmf	NB	NB	100	606	44	NB	16	294	NB
Hrk	NB	NB	>1000	3220	39	NB	24	312	1912
Noxa	NB	NB	>1000	NB	NB	NB	28	3284	NB
Puma	NB	NB	>1000	468	56	NB	24	2484	1753

NB-No Binding, N/A- Not Available.

Deerpox virus DPV022 is another apoptosis inhibitory Bcl-2 gene identified in capripoxviruses and does not feature obvious BH motifs. DPV022 was shown to block intrinsic apoptosis by interacting with a highly selective subset of pro-apoptotic protein Bak, Bax [121], and Bim. The structure of DPV022 revealed a domain swapped Bcl-2 fold [74], which has previously been seen in vaccinia and variola virus F1L [38,39,54].

### 1.6. Avipoxviruses

Avipoxviruses are a group of prominent pathogenic viruses among avian species, causing a slower growth in birds [122]. Among the sequenced genomes of *avipoxviridae genus,* putative Bcl-2 proteins of two members have been characterized, FPV039 from fowlpox virus (FPV) and CNP058 from canarypox virus (CNPV). Both of these proteins adopt the conserved monomeric Bcl-2 fold with seven alpha helices [119,120]. FPV039 is able to suppress the host apoptotic machinery by engaging with all pro-apoptotic Bcl-2 proteins (Table 1) [119], and the FPV039:Bax interaction prevents Bax oligomerization and mitochondrial pore-formation [123]. In contrast, CNP058 interacts with a distinct set of BH3 only proteins and did not show any detectable affinity toward Bok, Bad, or Bik (Table 1) [120]. Thus, both FPV039 and CNP059 potentially block host apoptosis by sequestering Bim and direct interactions with cellular Bak and Bax [119,120].

## 2. Extrinsic Apoptosis Inhibition

The extrinsic apoptosis pathway is initiated through the interaction of the death ligand (Fas/TNF) with their corresponding death receptors (Fas receptor/TNF receptor1) [124], as outlined in the introduction (Figure 2). The Fas ligand regulates the activation of apoptosis of natural killer cells and peripheral lymphocytes and is important for the induction of apoptosis in virally infected cells [14]. Binding of death-inducing ligands to a death receptor triggers a structural rearrangement of the receptor, accompanied by trimerization, which initiates the activation of pro-caspase-8 through interactions with adaptor proteins FADD or TRADD [14,125], which ultimately leads to the activation of the caspase cascade (Figure 2). Activation of the intrinsic pathway occurs via caspase-8 cleavage of Bid [126]. Poxviruses have been shown to encode multiple inhibitors of extrinsic apoptosis and this includes caspase inhibitors, tumor necrosis factor (TNF) homologs, and death effector proteins to evade the host immune system [7,35] (Figure 2). In general, these methods of apoptosis modulation have been less studied at a molecular level than the Bcl-2 related genes.

### 2.1. Poxvirus Encoded Caspase Inhibitors

Cytokine response modifier A (CrmA) is a 38 kDa protein that was the first identified caspase inhibitor encoded by cowpox virus (CPXV) and shares significant sequence homology with serine protease inhibitors (serpin). CrmA is expressed during the early infection phase [127] and suppresses the activity of both cysteine and serine proteases [128,129]. CrmA inhibits Granzyme B, a serine protease secreted by cytotoxic T lymphocytes that activates the caspase cascade [130]. In particular, CrmA inhibits caspase-1 and caspase-8 [128] with caspase-8 being vital for both extrinsic and mitochondrial apoptosis (Figure 2) [131]. CrmA interacts with caspase-1 and downregulates the production of mature pro-inflammatory cytokines such as interlukin-1β (IL-1β), which play an important role against poxvirus infections [132]. It has also been shown that CrmA knockout CPXV in embryonated chicken eggs produce inflammatory lesions with a lower level of CPXV replication compared to wild type virus. However, wild type virus produces non-inflammatory lesions upon infection [133].

Vaccinia virus encodes B13 (SPI2), a homolog of CPXV CrmA, with which it shares 92% sequence identity. B13 was shown to function similarly to CrmA and inhibits the action of several initiator caspases, various apoptotic stimulators including FasL, TNFα, and staurosporine to block apoptosis [56,134]. Compared to other known apoptotic inhibitors encoded by VACV such as F1L, N1L, or Golgi anti-apoptotic protein vGAAP, VACV B13 was the most virulent inhibitor of both extrinsic and mitochondrial apoptosis [135]. VACV B22R (SPI1) is a second serpin like protein that shares 44% sequence identity with VACV B13 and is found in all orthopoxvirus genuses, whereas the B13 gene was found only in a limited number of strains of vaccinia virus and was not detected in VACV Copenhagen, Lister, Tashkent, and Tian Tan [136]. B22R is important for viral replication with a B22R KO leading to a reduction of viral replication by two logs in A549 cells, with those cells undergoing TNF mediated apoptosis [137]. Both of these proteins were expressed in early infection cycle [136]. SPI3 is the third serpin like protein encoded by the vaccinia virus, which is not important for virulence, but was shown to inhibit cell fusion during infection [138]. MYXV was also shown to encode three serpin inhibitor homologs called SERP-1, SERP-2, and SERP-3 [139]. SERP-1 is an essential element of MYXV virulence [57], whereas SERP-2 functions by inhibiting caspase-1 and granzyme B [140]. However, the function of SERP-3 remains to be clarified [141]. However, previous studies have reported that exchanging CPXV CrmA and MYXY SERP2 between different poxviruses showed that they are not completely interchangeable even though they have some functional similarities such as both suppressing caspase-1 and granzyme-B [142].

### 2.2. Tumor Necrosis Factor (TNF) Homologs Encoded by Poxvirus

The TNF receptor is vital for initiating the extrinsic apoptosis pathway [14] (Figure 2). Poxvirus encoded anti-apoptotic proteins mimic the action of TNF receptor or bind TNF receptors to inhibit their function [143]. The first identified poxviral TNF receptor like proteins were myxoma virus M-T2 and shope fibroma virus S-T2 [144]. Both proteins have been shown to mimic the action of TNF and block the TNF induced extrinsic apoptosis in the host [145]. To date, five orthopoxvirus encoded TNF receptor homologs have been reported including CrmB, CrmC, CrmD, CrmE, and ectromelia virus encoded CD30 homolog vCD30 [7,146,147]. vCD30 was shown to interact with CD30L and block the interaction with CD30 [146]. Cowpox virus was also shown to feature four unique TNF receptor mimics (CrmB, CrmC, CrmD, CrmE) and a distinct CD30 homolog [7,147], where CD30 is a member of the TNFR family of proteins [146,148]. Crystallographic data showed that CPXV CrmE interacts with TNF [149]. Similarly, VARV encoded CrmB and its interactions with TNF have been widely studied and affinity measurements were reported. VARV CrmB binds to human TNF with similar affinity (0.28 nM) compared to human TNFR2 (0.3 nM) [150], but is a more efficient inhibitor of human TNF-induced cytotoxicity. More recent work has shown that tanapox virus encoded 2L is a novel TNF binding protein, which does not show significant sequence homology to any cellular proteins. TANV 2L bound human TNF with very high specificity and affinity (43 pM) and blocked the subsequent activation caspase cascade of cell death [151]. 

### 2.3. Death Effector Domain (DED) Homologs Encoded by Molluscum Contagious Virus

Molluscum contagiosum virus (MCV) is the only known member of the mulluscipoxvirus genus and one of two human specific pox viruses aside from variola virus. MCV infection is commonly seen in the young and causes a skin rash [152]. This virus has been shown to utilize an alternative approach to block death receptor mediated apoptosis. MCV encodes two proteins, MC159 and MC160, which feature sequence homology to the death effector motif of adaptor proteins TRADD and FADD, and initiator caspase [153,154]. Both MC159 and MC160 contain two death effector domains (DED), which are important for protein–protein interactions in the apoptotic signaling pathway [153,155] and also called viral FLICE (FADD-like IL-1_converting enzyme)-like inhibitory proteins (vFLIP). These proteins are important not only in apoptosis activation, but also in necroptosis, NF-κB and interferon signaling [156,157,158]. These DED domain homologs can be seen in several pro-apoptotic proteins including FADD and pro-caspase-8, and both MC159 and MC160 mimic the pro-caspase-8 domain structure of a tandem DED domain rather than the single DED domain as seen in FADD [156,159]. This suggests that MC159 and MC160 could regulate the extrinsic apoptosis pathway through the interactions of DED domain as similar to that observed in FasL, FADD, and pro-caspase-8 assembly. 

MC159 is a 241 residue protein encoded by MCV and expressed during early infection stage. MC159 is crucial to block host TNF-α/Fas induced extrinsic apoptosis pathway via the interactions of two N-terminal DED sequence motifs [153,156,159] Both of these DED motifs contain the well conserved consensus “RxDL” motif as previously seen in other DED containing proteins such as MC160, pro-caspase-8, and FADD. The crystal structure of MC159 revealed that two DED motifs of MC159 were tightly associated by hydrophobic interactions [160,161] and the Arg (R) and Asp (D) residues of the conserved DED motif cooperated to create a network of hydrogen bonding interactions [160,161]. Interestingly, site directed mutagenesis of any of these two residues into Ala (A) resulted in the loss of function [160,162]. Additionally, MC159 was shown to engage with DED motifs of procaspase-8 and FADD [154,159]. The expression of MC159 during viral infection could inhibit the development of death effector filaments and capping to suppress the activation of caspase-8 [163,164]. The C-terminal region of MC159 contains three conserved TRAF3 interacting sequence motifs (with a consensus sequence PxQxS/T, where x is any residue), which are crucial for recruiting both TRAF2 and TRAF3 to form the DISC complex. However, a TRAF interacting region deletion mutant of MC159 partially undergoes Fas induced apoptosis in Jurkat cells. This suggests that MC159 could inhibit Fas induced apoptosis in both a TRAF dependent and independent manner [165]. However, in-vitro affinity measurements or structural data for these interactions are not available.

The role of MC159 in MCV virulence is poorly understood due to a lack of good model systems to study this activity. Previous studies performed using recombinant VACV with the CrmA deletion mutant that expresses MC159 blocked the Fas-mediated caspase-3 activation and subsequent caspase-8 activation, hence suppressing apoptosis [159]. This study suggests that VACV and MCV infections utilized a similar kind of apoptosis inhibition upon expressing MC159 [159]. A similar study was performed using a murine cytomegalovirus (MCMV) M36 deletion mutant (M36-MCMV encoded caspase-8 inhibitor) supplemented with MC159 and it was shown that in contrast to the MCMV MC36 deletion virus, MC159 expressing recombinant MCMV virus blocks TNF-mediated apoptosis [166]. This suggests that MC159 acts in a similar manner to that previously reported in an ectopic expression system [166]. 

MC160 is the second DED containing protein expressed by MCV during infection, which comprises 371 amino acids and features a relatively long C-terminal extension compared to MC159 [156]. The overall sequence identity of the two DED motifs of MC160 was 45% and 33%, respectively, compared to MC159 [159]. No structural data are available for MC160, but recent homology modeling using MC159 has been reported and both MC159 and MC160 are predicted to share key hydrogen bonding interacting residues [153] and therefore the structure of MC160 is likely to be similar to that in MC159. Similar to MC159, MC160 was also shown to interact with procaspase-8 and FADD through the conserved DED region [158]. However, expression of MC160 does not interfere with the extrinsic apoptotic pathway when transfected during VACV infection [159]. Interestingly, transfection and expression of MC160 in VACV infected cells does not block caspase-3 and caspase-8 [159]. The sequence of MC160 was shown to contain a caspase-8 cleavage site, thus rendering it susceptible to caspase-8 cleavage [159]. However, co-expression of MC160 with MC159 was shown to suppress the caspase cleavage of MC160 [159]. Additionally, both MC159 and MC160 were shown to inhibit NF-κB activation through degradation of iκBβ [157], and MC159 has been reported to block the PKR mediated NF-κB activation and PKR induced apoptosis [153,155,167]. Combined, these studies show the capacity of poxviruses to utilize multiple alternative strategies to inhibit host extrinsic apoptosis pathways to aid virus replication.

## 3. Poxvirus Encoded Indirect Influencers of Apoptotic Signaling

The sections above describe poxvirus involvement in the direct inhibition of host cell apoptosis. However, poxviruses also encode numerous other inhibitory proteins that are indirectly involved in the inhibition of either intrinsic or extrinsic apoptosis. These include poxvirus expression of Golgi anti-apoptotic protein (GAAP) [58,168], Cu–Zn–superoxide dismutase and double stranded RNA (dsRNA) induced apoptosis [7] (Figure 2). These inhibitors underscore the fact that viruses have evolved diverse repertoires of cell death inhibitors that may act directly or indirectly on the different apoptosis mechanisms in host cells, in order to ensure their own survival and proliferation, as summarized in Table 2.

### 3.1. vGAAP

Camelpoxvirus (CMV) encoded 6L is a 237 residue protein homologous to cellular Golgi anti-apoptotic protein, and shares 73% identity with its cellular counterpart [168,169]. This protein consists of multiple transmembrane domains and expresses during the early infection cycle where it was primarily localized to the Golgi apparatus [168]. Genome analysis of VACV strains reported that vGAAP was only present in a few VACV strains including Evans, Lister, and USSR [168]. It has been reported that transient expression of VACV GAAP in cells could block both mitochondrial and extrinsic apoptosis induced by various stimulus such as overexpression of apoptosis regulator Bax, Fas antibodies, TNFα, doxorubicin, and staurosporine [168]. Mice infected with VACV GAAP KO virus showed a higher number of viral particles in their body and severe infection symptoms [168]. Interestingly, both cGAAP and vGAAP have been shown to form ion channels in the Golgi apparatus and contribute to passive leak of Ca^2+^ [58,170]. This leakage will reduce the Ca^2+^ concentration in the Golgi apparatus and therefore affect apoptosis induced by Ca^2+^ [58,170]. vGAAP was the first reported protein encoded by poxviruses that could form ion channels [170]. Together, these data suggest that vGAAP plays an important role during VACV infection by blocking both extrinsic and intrinsic apoptosis.

### 3.2. Cu-Zn-Superoxide Dismutase Homologs

Cellular Cu–Zn–superoxide dismutase (SOD) is a multifunctional homodimeric protein that is important to catalyze superoxide radical dismutation [171] and ultimately protects cells from oxidative stress [61]. Previous studies reported that SOD has a function in innate immune responses against bacteria or viral infections, since the superoxide radical is one of the most powerful toxic molecules produced by immune cells to kill bacteria or viral infected cells [172]. Numerous poxviruses that encode well-conserved structural homologs of Cu–Zn–SOD have been identified including molluscum contagiosum virus (MCV) [173], leporipoxvirus [61,174], vaccinia virus [175], and Amsactamoorei entomopoxvirus [176]. Interestingly, most of these poxviruses encoded SOD are catalytically inactive because of the lack of essential regions in their sequence [61], whereas accumulation of superoxide in cells during poxviral infection has been proposed to have an anti-apoptotic effect and tumorigenic function [61]. Among those poxviruses that encode Cu–Zn–SOD, MCV and leporipoxvirus SOD have been widely studied. The MCV Cu–Zn–SOD homolog, MC163 localized into mitochondria with N-terminal hydrophobic region and expression of MC163 was shown to block apoptosis induced by staurosporine or TNF/cyclohexamide in HeLa cells and to inhibit intrinsic apoptosis by preventing mitochondrial outer membrane permeabilization [173]. MC163 was shown to inhibit staurosporine induced Caspase-3 activation [173]. This suggests that MC163 plays a crucial role in MCV infection by blocking host intrinsic apoptosis. Two members of the leporipoxvirus family were shown to encode a Cu–Zn–SOD homolog: MYXV M131 and Shope fibroma virus (SFV) S131, which are 96% identical to each other and are expressed in later stages of the infection [177]. Neither MYXV or SFV Cu–Zn–SOD homologs showed any catalytic activity [174]. Expression of MYXV M131 inhibits staurosporine and Fas induced apoptosis in Jurkat cells [177], while the gene knockout of MYXV M131 could not protect cells undergoing apoptosis initiated by the Fas ligand [177]. Similarly, knockout of the SFV S131 gene from virus produced fairly small tumors in vivo, whilst wild type S131 showed significantly larger tumors [177]. Interestingly, Amsactamoorei entomopoxvirus encoded Cu–Zn–SOD, AMV255, was shown to have catalytic activity and was the first reported active Cu–Zn–SOD found in poxviruses. Deletion of the AMV255 gene prevented viral proliferation in cell culture [176]. Similarly, Cu–Zn–SOD like protein found in vaccinia virus A45R, is a highly conserved protein among orthopoxviruses, whilst it also does not show any catalytic activity and no effect on viral replication [175]. Overall, poxvirus encoded SOD homologs employ multiple mechanisms to contribute to host defense evasion, thus representing an important tool in the viral armory. 

### 3.3. Poxvirus Inhibition of Double Stranded RNA (dsRNA) Induced Apoptosis

Host cells have developed multiple proteins that sense and counter viral dsRNA and activate apoptosis, type1 interferon (INF-1) synthesis, and inhibition of protein synthesis in response to viral infection [178]. The presence of dsRNA in a cell is a unique signature of viral infection and a vital pathogen associated molecular pattern (PAMP) that is identified by intracellular and extracellular molecules [179]. dsRNA synthesis is a post infection event and poxviruses have evolved to prevent this happening during the lytic cycle of infection [178]. However, virus produce a notable amount of dsRNA in late infection, which potentiates numerous innate immune responses such as apoptosis through FADD and procaspase-8 activation [59,180].

### 3.4. Poxvirus Encoded E3-PKR Inhibitor of Apoptosis

Host innate immune system pattern recognition receptor (PRRs) molecules play a remarkable role against invading pathogens to initiate a powerful antiviral response. Poxviruses have evolved to counteract the action of PRRs by mimicking the action of host protein kinase R (PKR). PKR are activated by dsRNA through the induction of IFN and are important for the inhibition of protein synthesis through the phosphorylation of eukaryotic translation initiation factor 2α (eIF2 α) [181]. The VACV encoded E3L gene is responsible for the expression of the E3 protein in early infection that interacts with dsRNA at its C-terminal binding domain [182,183] and suppresses the activation of PKR preventing eIF2α phosphorylation and subsequent dsRNA induced apoptosis [59,184]. Genetic deletion of E3 restored apoptosis [59]. The results of these experiments establish that VACV E3 plays an important role during viral infection and protects cells from initiating apoptosis. Several other poxviruses encode putative E3 homologs including myxoma virus, MYXV M029, and swinepox virus, SPV032, which inhibit dsRNA induced PKR activation [185]. MYXV M029L gene knockout evidence showed that European rabbits are highly resistant to infection by MYXVΔM029L and do not show any sign of myxomatosis and PKR activation [186]. Furthermore, MYXV M029L was shown to interact with PKR in immunoprecipitation experiments [186]. However, affinity measurements of this interaction have not been reported. It has been proposed that MYXY M029L functions similar to VACV E3 to downregulate PKR activation, protecting virus infected cells from undergoing apoptosis and thus enabling viral replication. In contrast, E3 homologs identified in sheeppox virus or yaba monkey tumor virus do not show any PKR suppression activity [185]. This suggested that poxvirus encoded E3 are important for interactions with dsRNA and subsequent apoptosis inhibition by contributing to multiple aspects of virulence during the infection.

**Table 2:** Summary of poxvirus encoded inhibitors of apoptosis.

Genus	Virus	Protein Type	Protein	Function
*Orthopoxviridae*	VACV	Bcl-2 like	F1L	Pro-survival [38,54]
			N1L	NF-κB inhibition [87]
			A46	NF-κB inhibition [90]
			A49	NF-κB inhibition [102,104]
			A52	NF-κB inhibition [95]
			B14	NF-κB inhibition [95]
			B15	NF-κB inhibition [187]
			K7	NF-κB inhibition [187] IFN signaling [100]
		Serpin	B13 (SPI-1)	Caspase inhibition [56,134,136]
			B22 (SPI-2)	Caspase inhibition [136,137]
			SPI-3	Caspase inhibition [138]
		vTNFR	CrmB	Mimic TNFR1/2 [188]
			CrmC	Mimic TNFR1/2 [188]
			CrmE	Mimic TNFR1/2 [188]
		SOD Homolog	A45	Inactive SOD like virion [175]
		PKR inhibitor	E3	Binds to PKR and inhibit activation of PKR [185]
		Decapping enzymes	D9/ D10	PKR activation inhibitor [189]
		Ankyrin-repeat protein	M1L	Apoptosome inhibitor [105]
	VARV	Bcl-2 like	F1L	Pro-survival [39]
		vTNFR	CrmB	Mimic TNFR1/2 [150]
	CPXV	Serpin	CrmA	Caspase inhibition [190,191]
		vTNFR	CrmB	Mimic TNFR1/2 [192]
			CrmC	Mimic TNFR1/2 [192]
			CrmD	Mimic TNFR1/2 [192]
			CrmE	Mimic TNFR1/2 [149]
			vCD30	Mimic TNFR1/2 [147]
	CMLV	vGAAP	6L	Anti-apoptotic [58]
	ECTV	Bcl-2 like	EMV025	Pro-survival [84]
		vTNFR	vCD30	Mimic TNFR1/2 [146]
*Leporipoxviridae*	MYXV	Bcl-2 like	M11L	Pro-survival [37]
		Serpin	SERP1	Caspase inhibition [57]
			SERP2	Caspase inhibition [140]
			SERP3	Caspase inhibition [141]
		vTNFR	M-T2	Mimic TNFR1/2 [145]
		SOD homolog	M131	SOD induced anti-apoptosis [174]
		E3 homolog	M029	PKR activation inhibitor [186]
	SFV	vTNFR	T2	Mimic TNFR1/2 [145,193]
		SOD homolog	S131	SOD induced anti-apoptosis [174]
		E3 homolog	SPV032	PKR activation inhibitor [185]
*Yatapoxviridae*	TANV	Bcl-2 like	16L	Pro-survival [110]
		vTNFR	2L	Mimic TNFR1/2 [151]
*Parapoxviridae*	ORFV	Bcl-2 like	ORFV125	Pro-survival [113,114,115]
*Capripoxviridae*	SPPV	Bcl-2 like	SPPV14	Pro-survival [117,118]
*Cervidpoxviridae*	DPV	Bcl-2 like	DPV022	Pro-survival [74]
*Avipoxviridae*	FPV	Bcl-2 like	FPV039	Pro-survival [119,123]
	CNP	Bcl-2 like	CNP058	Pro-survival [120]
*Molluscipoxviridae*	MCV	SOD homolog	MC163	SOD induced anti-apoptosis [173]
		Seleno protein	MC066	Inhibit H_2_O_2_ and UV induced apoptosis [194]
		vFLIP	MC159	Inhibitor TNF-α/FasL induced apoptosis [156,157,159,167]
			MC160	Inhibitor TNF-α/induced NF-κB inhibition [157,158]

## 4. Concluding Remarks

Initiation of apoptosis is a crucial frontline defense mechanism for host cells to repel invading pathogens. With their large genomes, poxviruses have developed a diverse array of anti-apoptotic strategies to block host cell suicide mechanisms. As outlined above, poxviruses have evolved both direct and indirect inhibition mechanisms of host apoptosis for successful infections. The direct apoptosis inhibition includes viral Bcl-2 mediated mitochondrial apoptosis and caspase cascade inhibition and deactivation of death receptor molecules. Indirect inhibition includes the inhibition of dsRNA induced apoptosis, mimicry of Golgi anti-apoptotic protein, and Cu–Zn–SOD inhibition. These features highlight the sophistication of poxviruses in targeting apoptosis. Currently, there is a paucity of direct interaction measurements or structural data to indicate how these molecules engage with crucial host signaling proteins. Furthermore, it is clear that even when binding data are available, there are multiple modes of apoptosis inhibition by the viral Bcl-2 homologs. In addition to the numerous described poxviral encoded proteins used to disarm the plethora of apoptosis-associated host responses to viral infection, a number of poxviral modulators of another critical host defense system exist that counter interferon (IFN)-based responses. These have been critically reviewed recently [9,10], and consequently, we did not include IFN regulatory mechanisms in this review.

While recent studies on the structure and interactions of poxvirus encoded anti-apoptotic proteins have attempted to provide a more detailed understanding of apoptosis inhibition in poxviruses, many gaps remain in our understanding of their modulation of host cell apoptosis.
